# An mRNA assay system demonstrates proteasomal-specific degradation contributes to cardiomyopathic phospholamban null mutation

**DOI:** 10.1186/s10020-021-00362-8

**Published:** 2021-09-08

**Authors:** Eduarde Rohner, Nevin Witman, Jesper Sohlmer, Erwin De Genst, William E. Louch, Makoto Sahara, Kenneth R. Chien

**Affiliations:** 1 Department of Cell and Molecular Biology, Karolinska Institutet, Stockholm, Sweden; 2 Integrated Cardio Metabolic Center, Department of Medicine, Karolinska Institutet, Huddinge, Sweden; 3 Department of Clinical Neuroscience, Karolinska Institutet, Stockholm, Sweden; 4 Institute for Experimental Medical Research, Oslo University Hospital and University of Oslo, Oslo, Norway; 5 K. G. Jebsen Cardiac Research Center, University of Oslo, Oslo, Norway; 6 Department of Surgery, Yale University School of Medicine, New Haven, CN USA

**Keywords:** Disease model, L39X, Modified mRNA, PLN

## Abstract

**Background:**

The human L39X phospholamban (PLN) cardiomyopathic mutant has previously been reported as a null mutation but the detailed molecular pathways that lead to the complete lack of detectable protein remain to be clarified. Previous studies have shown the implication between an impaired cellular degradation homeostasis and cardiomyopathy development. Therefore, uncovering the underlying mechanism responsible for the lack of PLN protein has important implications in understanding the patient pathology, chronic human calcium dysregulation and aid the development of potential therapeutics.

**Methods:**

A panel of mutant and wild-type reporter tagged PLN modified mRNA (modRNA) constructs were transfected in human embryonic stem cell-derived cardiomyocytes. Lysosomal and proteasomal chemical inhibitors were used together with cell imaging and protein analysis tools in order to dissect degradation pathways associated with expressed PLN constructs. Transcriptional profiling of the cardiomyocytes transfected by wild-type or L39X mutant PLN modRNA was analysed with bulk RNA sequencing.

**Results:**

Our modRNA assay system revealed that transfected L39X mRNA was stable and actively translated in vitro but with only trace amount of protein detectable. Proteasomal inhibition of cardiomyocytes transfected with L39X mutant PLN modRNA showed a fourfold increase in protein expression levels. Additionally, RNA sequencing analysis of protein degradational pathways showed a significant distinct transcriptomic signature between wild-type and L39X mutant PLN modRNA transfected cardiomyocytes.

**Conclusion:**

Our results demonstrate that the cardiomyopathic PLN null mutant L39X is rapidly, actively and specifically degraded by proteasomal pathways. Herein, and to the best of our knowledge, we report for the first time the usage of modified mRNAs to screen for and illuminate alternative molecular pathways found in genes associated with inherited cardiomyopathies.

**Supplementary Information:**

The online version contains supplementary material available at 10.1186/s10020-021-00362-8.

## Background

In the heart, calcium plays a crucial role in regulating contractile force and rate (Davlouros et al. 2016). Impairment of calcium regulatory genes and calcium dysregulation have been associated as a causative and negative indicator of several cardiomyopathies and progression towards heart failure (Davlouros et al. 2016; Deftereos et al. 2016; Marks 2013). Within the complex and dynamic calcium handling machinery, the 52 amino acid protein phospholamban (PLN), acts as a reversible inhibitor of the sarco-endoplasmic reticulum calcium ATPase 2a (SERCA) (Marks 2013; Frank and Kranias 2000). Given SERCA’s central role in inducing cardiac relaxation in a diastole phase by recycling calcium into the sarcoplasmic reticulum, PLN has proven to be an important piece of the puzzle to elucidate mechanisms of human calcium regulation (Marks 2013; Frank and Kranias 2000; Kranias and Hajjar 2017).

Throughout the last two decades, a handful of naturally occurring PLN mutants have been uncovered in humans (Opbergen et al. 2017). Despite varying sizes and geographical distribution of these mutant PLN patient populations, there is a similar strong association between PLN mutations and cardiomyopathic phenotypes led by chronic calcium misregulation (Kranias and Hajjar 2017, 2012; Opbergen et al. 2017).

**Fig. 1:**
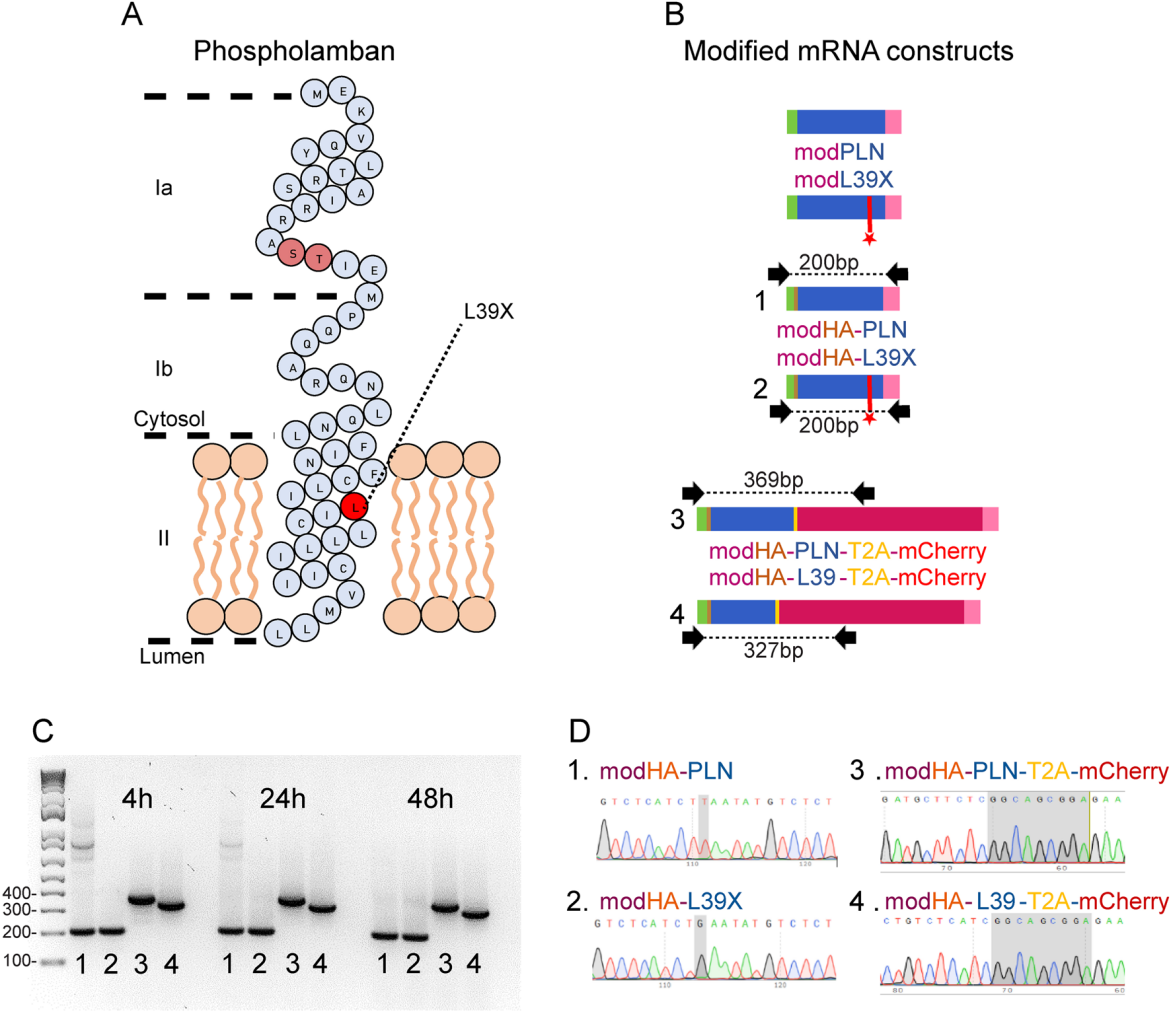
PLN modified mRNAs overview and mRNA in vitro stability assay. **A** Schematic representation of PLN protein monomeric structure and of its regulatory domain Ia, Ib and transmembrane domain II, location of regulatory phosphorylated amino acid (pink) and location of L39X mutation (red). **B** Schematic representation of the different modified mRNA constructs. 5’UTR and analogue cap (green), PLN CDS (dark blue), 3’UTR and Poly-A trail (pink), HA tag (orange), T2A sequence (yellow), mCherry (red), G-> T mutation (red star), black arrows indicate FWD and REV cDNA PCR Primers. **C** PCR amplified cDNA gel electrophoresis showing the presence of modHA-PLN (1), modHA-L39X (2), modHA-PLN-T2A-mCherry (3), modHA-L39-T2A-mCherry (4) transfected modified mRNA after 4 h, 24 h, and 48 h respectively. **D** Sanger sequencing of PCR amplified cDNA showing the expected wild-type and mutated sequences of the transfected modRNA constructs

While only heterozygous carriers have been reported for most of these pathogenic mutants, the L39X variant stands out as being described as a functionally null mutant (Haghighi et al. 2003). The T to G nonsense substitution of the 116th nucleotide in this variant converts the 39th Leucine into a premature stop codon interrupting the protein’s normal transmembrane domain (Fig. 1A).

Interestingly, both L39X plasmid overexpression in HEK293 cells and explanted hearts from L39X homozygous patients showed a lack of PLN protein (Haghighi et al. 2003). Further functional assessment showed no inhibitory effect on SERCA’s activity from L39X overexpression in rat cardiomyocytes and in AAV-293 cells compared to wild-type (WT) PLN (Haghighi et al. 2003; Kelly et al. 2008). Therefore, the L39X mutant was concluded to lead to a PLN-null mutation (Haghighi et al. 2003). Importantly, this mutant illustrated a clear mechanistic difference in between mouse and human cardiac calcium regulation, as the PLN ablated mouse showed no chronic detrimental effects contrasting with the severe cardiomyopathic phenotype seen in both homozygous L39X human carriers (Haghighi et al. 2003; Slack et al. 2001). Regardless, no clear mechanism explaining the lack of detectable protein has since been established.

Cellular protein degradation mechanisms including the proteasome and the lysosome have been associated to heart pathologies (Schlossarek et al. 2014; Gilda and Gomes 2017; Wang and Robbins 2014). Recently, other well studied truncated cardiac proteins have been shown to be actively targeted by either proteasomal or non-sense mediated decay pathways (Sarikas et al. 2005; Seeger et al. 2019; Geiger et al. 2008). Interestingly, one of these studies has also shown that the expression of those truncated proteins is correlated to an impaired cellular degradation homeostasis which could further induce cardiomyopathies in models of iPS-derived cardiomyocytes (Seeger et al. 2019). Therefore, identifying the mechanism by which the L39X variant’s expression is impaired or degraded could have important implications in understanding the observed phenotype in human carriers and our overall understanding of human calcium regulation in the heart.

Disease modelling currently relies on combining stem cells, genetic editing and plasmid/viral overexpression technologies, and these approaches have helped to uncover many biological mechanisms (Prondzynski et al. 2019; Sumer et al. 2020; Lodola et al. 2016; Pasquale et al. 2013; Moretti et al. 2010). Recently, chemically modified mRNA (modRNA) has surged as a novel therapeutic modality (Gan et al. 2019; Baden et al. 2020; Richner et al. 2017; Polack et al. 2020). An mRNA-based platform aimed at regenerative medicine has become increasingly valuable thanks to its capacity to efficiently and reliably express therapeutic levels of proteins in vivo, while avoiding safety caveats and technical limitations often associated with gene therapy (Gan et al. 2019; Baden et al. 2020; Richner et al. 2017; Polack et al. 2020; Boo and Kim 2020). However, modRNA has thus far scarcely been employed as a disease modelling tool to investigate translational and/or post-translational mechanistic defects (Zhu et al. 2019; Karadagi et al. 2020).

In this report, we investigated the mechanism by which the L39X phospholamban mutant produces the observed null phenotype. We established an mRNA assay system employing human embryonic stem cell (hESC)-derived cardiomyocytes, modRNA and chemical inhibitors to showcase that the L39X mutant is rapidly degraded by the proteasome.

## Materials and methods

### Generation of modified mRNA constructs

All linearized DNA plasmids used as templates for modRNA synthesis incorporate a 5’ and 3’ untranslated region (UTR) and poly-A tail as previously described (Warren et al. 2010). The open reading frame sequence used in our 3 different constructs contain the coding sequence for WT human PLN (CCDS5120.1). L39X versions of these plasmids were generated using a site-directed-mutagenesis kit (Agilent) substituting the 116th Thymine to a Guanine. In vitro synthesis of modRNA was accomplished through T7 RNA polymerase mediated transcription as previously described (Yu et al. 2019). All modRNAs were synthesized using the chemically modified nucleotides, 5’-methylcytidine and pseudouridine in place of cytosine and uridine. RNA was quantified and quality controlled by nanodrop (Thermo Scientific) and by Bioanalyzer (Agilent) respectively.

### Transfection of modified mRNA

ModRNAs were transfected in vitro using RNAiMax transfection reagent (Thermo Scientific). OptiMEM basal media (Thermo Scientific) was used to dilute RNAiMax and modRNAs. 2.5 µL of transfection reagent was used per 500 ng of modRNA. Lipid-RNA complexes were generated by mixing and incubating diluted modRNA and transfection reagent for 15 min at room temperature. The mixture was then added to the cells for 4 h at 37 °C before performing a media change. Similar volumes of carrier only RNAiMax were used as negative controls. Cells were further analysed 24 h–32 h post-transfection.

### Cell culture, differentiation and re-seeding

H9 hESCs were cultured in E8 basal media (Thermo Scientific) and maintained on Matrigel (Corning) coated 6-well TC dishes. Cells were passaged every 3–4 days with Versene. Wnt signaling modulation based cardiac differentiation protocol was adapted from a previously published protocol (Lian et al. 2013). Day 23 differentiated cardiomyocytes were harvested using 0.25% Trypsin–EDTA and neutralized in RPMI b27 + supplemented with 20% FBS. Re-suspended cells were then passed through a 100 µm mesh to remove residual clumps. 2 × 10^6^ cardiomyocytes were re-seeded in RPMI b27 + , 10%FBS and Y-inhibitor (5 µM) in RPMI b27 + on Matrigel coated 12well TC plate (with or without coverslips). RPMI b27 + media was changed every 48 h. Day 28.5–30 cardiomyocytes were used for the performed experiments.

### RNA extraction and PCR

Total RNA was harvested using RNAeasy kit (Thermo Scientific). cDNA was synthesized from 1 µg of harvested total RNA, using Revertaid reverse transcription kit (Thermo Scientific). cDNA from each sample was used as a template for PCR amplification using primers specific for modRNA sequences. PCR amplicons were separated on a 2% poly-acrylamide gel.

### Immunofluorescence

Cells were fixed using 10% Formalin (Sigma) for 10 min and blocked in 1% BSA in PBST (0.1% Tween) for 1 h at room temperature. Primary antibody staining was performed overnight at 4 °C and secondary antibodies incubated for 1 h at room temperature in 1% BSA, PBST (0.2% Tween). Primary and secondary antibodies were used with the following dilutions: 1:500 PLN (Abcam, ab2865), 1:500 SERCA (Thermo Scientific, MA3-910), 1:300 HA (CST, C29F4), 1:300 cTNT (Abcam, ab10214), 1:500 α-Actinin (Sigma, A7811), 1:750 Alex-Fluor 488, 594 and 647 (Thermo Scientific). All images were acquired and analysed on a Zeiss LSM700 confocal microscope and imaging software.

### Immunoblotting

Protein lysate concentrations were measured by BCA protein assay kit (Thermo Scientific). 30 µg of protein lysates were loaded on a 12% Bis–Tris gel in MES buffer (Thermo Scientific). The gel was transferred to a 0.2 µm nitrocellulose membrane (Biorad) and blocked with 5% milk in TBST (0.1% Tween) for 1 h before being probed with primary antibodies 1:2000 GAPDH-HRP (CST, 8884S), 1:1000 HA (CST, C29F4), 1:1000 LC3B (CST, 3868), 1:1000 Ubiquitin (CST, 3933), 1:3000 mCherry (Thermo Scientific, PA5-34974) at 4 °C overnight. Each membrane was incubated with 1:2000 Anti-Rb-HRP conjugated secondary antibody (CST, 7074) for 2 h at room temperature. The membranes were washed 5 × with TBST after primary and secondary stainings and incubated with femto-plus ECL reagent (Thermo Scientific) for 5 min before imaging on a Chemidoc (Biorad).

### Statistical analysis

One-way Anova followed by Tukey–Kramer post-hoc test and Limma were used for statistical analysis. Statistical significance is defined by *P* < 0.05. All data shown in bar graphs are presented as mean ± SD.

### RNA sequencing and data analysis

cDNA libraries of harvested bulk RNA samples were generated using Illumina TrueSeq mRNA (Poly-A selection) kits and each library was sequenced at 150 bp paired-end on an Illumina NovaSeq 6000 S4 instrument to a depth of 2–4 × 10^7^ reads. The quality of the fastq-format sequenced data Raw was assessed using FASTQC, and raw reads were further trimmed and aligned onto human genome reference (hg38) using Cutadapt and STAR (Sahara et al. 2019). Transcript levels were quantified as fragments per kilo base of transcript per million mapped reads (FPKM). Further normalization and differential expression analysis were conducted using edgeR and Limma programs on R/Bioconductor (Sahara et al. 2019). Gene set enrichments analyses (GSEA) were performed on the GSEA software v4.1.0 (Broad institute).

## Results and discussion

We hypothesized that three possible mechanisms could result in a lack of detectable L39X variant protein: (1) The mutation could induce an actively degraded or unstable mRNA; (2) The mutation could affect the translational outcome of the mRNA or (3) The mutation could be responsible for driving an actively degraded and/or highly unstable protein.

### L39X mutation does not critically affect mRNA stability

Three different modRNA molecules were generated for both WT PLN and the mutant L39X sequences. First, we generated a native untagged mRNA sequence that was used for the RNA sequencing assay (modPLN and modL39X) (Fig. 1B and Additional file 1A). Next, we developed an mRNA sequence encoding a 5’ HA tag upstream of the WT and L39X PLN coding region (modHA-PLN and modHA-L39X). Finally, we produced mRNA sequences containing the previously mentioned 5’ HA tag, but we also included a 3’ T2A-mCherry fusion protein downstream of the WT and L39 PLN coding region (modHA-PLN-T2A-mCherry and modHA-L39-T2A-mCherry). Both tagged modRNAs were used in the cell imaging and protein expression assays (Fig. 1B and Additional file 1A). All of the employed modRNA molecules were designed and optimized for stability and translation efficiency. After in vitro synthesis and processing, all constructs produced similar RNA yield and purity, with no differences observed between the L39X and the WT PLN modRNA molecules on the Bioanalyzer (Additional file 1C).

We proceeded to validate the stability of the modRNAs in vitro. 500 ng of reporter PLN modRNAs were transfected in hESCs and total RNA was harvested at 4 h, 24 h and 48 h post-transfection (Additional file 1A). cDNA was generated, PCR amplified and sequenced using primers overlapping unique sequences from our constructs (Fig. 1B). The expected band sizes (200 bp HA constructs, 369 bp HA-PLN-T2A-mCherry and 327 bp HA-L39-T2A-mCherry) were clearly apparent at all time points similarly between WT PLN and L39X samples (Fig. 1C). Sanger sequencing of the PCR amplicon from the 24 h samples confirmed the correct WT PLN and mutated L39X sequences from the synthesized and transfected modRNA molecules (Fig. 1D). These data suggest the structural stability and integrity of the L39X mRNA is well-maintained and supports similar observations made in an explanted heart from a homozygous L39X patient (Haghighi et al. 2003).

### L39X modRNA is actively translated with negligible protein expressed

As we confirmed stability of the mutant PLN modRNAs in situ, we next sought to assess whether the L39X modRNAs were efficiently translated.

**Fig. 2:**
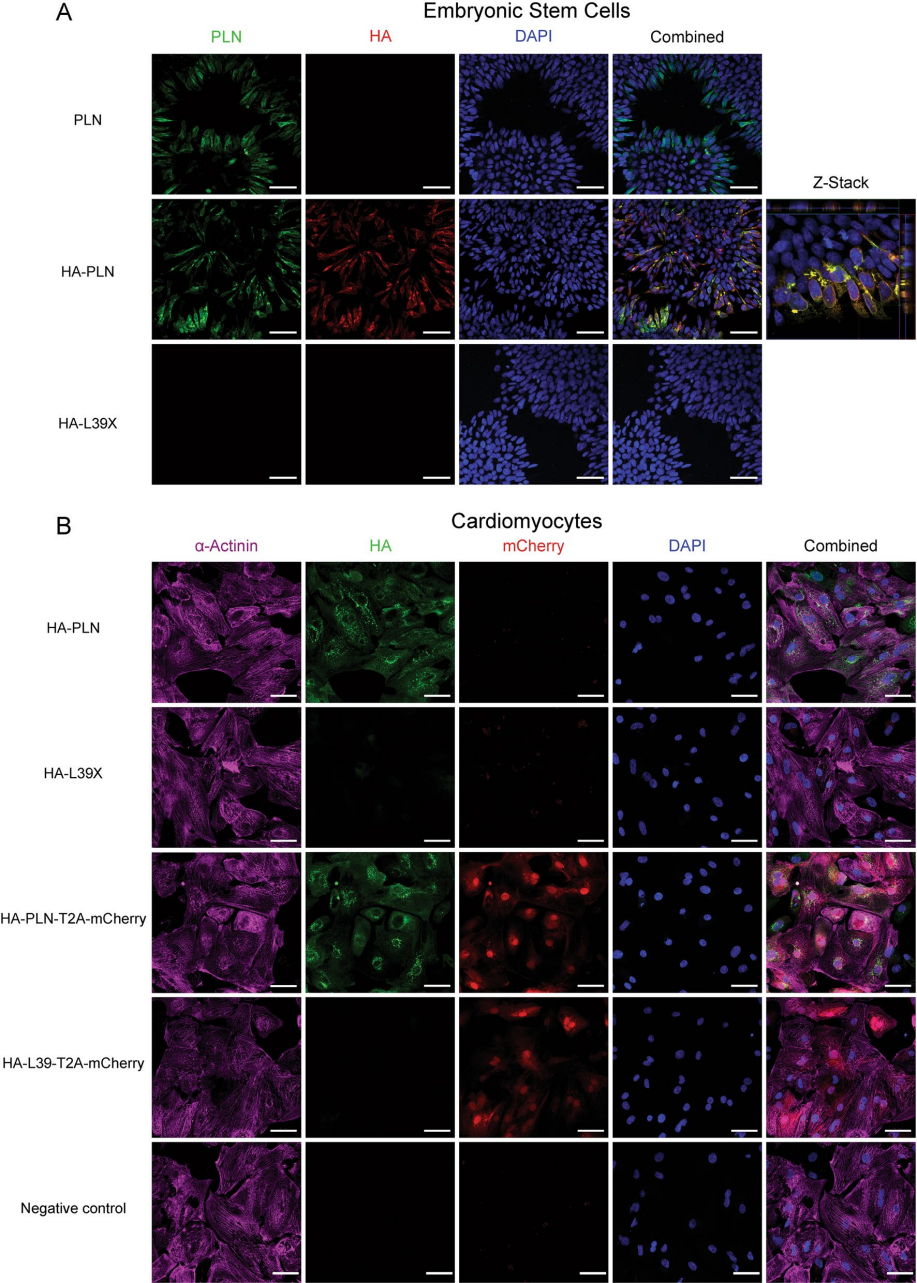
Expression of PLN modified mRNAs in hESC and differentiated cardiomyocytes. **A** Immunofluorescence images of modPLN (top), modHA-PLN (middle) and modHA-L39X (bottom) modRNA transfected hESCs. PLN (green), HA (red), DAPI (blue) indicating co-localization of PLN and HA signal (right panel). **B** Immunofluorescence images of modHA-PLN (first panel), modHA-L39X (second panel), modHA-PLN-T2A-mCherry (third panel), modHA-L39-T2A-mCherry (fourth panel) modRNAs transfected cardiomyocytes and negative control (last panel). α-Actinin (violet), HA (green), mCherry (red), DAPI blue. Scale bars = 50 µm

ModPLN and modHA-PLN mRNAs showed a similar robust perinuclear PLN protein expression which was co-localized with the HA signal in transfected hESCs (Fig. 2A).

As no clear protein signal could be seen in modHA-L39X transfected cells (Fig. 2A–B and Additional file 1D), we generated T2A-mCherry modRNAs to assess their active translation. The T2A sequence (GSG)-EGRGSLLTCGDVEENPGP acts as a ribosomal skipping sequence allowing the subsequent translation of two distinct proteins from a single polycistronic molecule. In this way it is possible to trace protein expression of a gene of interest without altering protein folding, stability or binding capabilities (Liu et al. 2017; Szymczak-Workman et al. 2012).

We proceeded in transfecting 500 ng of HA and T2A-mCherry modRNAs in day 29 hESC-derived cardiomyocytes (Fig. 2B). Widespread and comparable levels of mCherry protein were apparent 24 h post-transfection in both modHA-PLN-T2A-mCherry and modHA-L39-T2A-mCherry transfected cells indicating adequate transfection levels and similar translation efficiency of both modRNAs (Fig. 2B and Additional file 1D).

However, immunostaining from these cells showed a strong perinuclear HA signal only in modHA-PLN and modHA-PLN-T2A-mCherry transfected samples. In contrast, L39X equivalent transfections showed no clear HA signal (Fig. 2B and Additional file 1D). Therefore, we conclude that the modHA-L39-T2A-mCherry modRNA is actively translated in vitro, but the upstream HA-L39 sequence is not stably expressed as protein (Fig. 2B and Additional file 1D).

### Inhibition of the proteasome increases HA protein signal in modHA-L39X transfected cardiomyocytes

The combination of an abundant mCherry signal and a lacking HA signal stirred us to investigate whether the L39X mutant protein is actively and rapidly degraded.

**Fig. 3:**
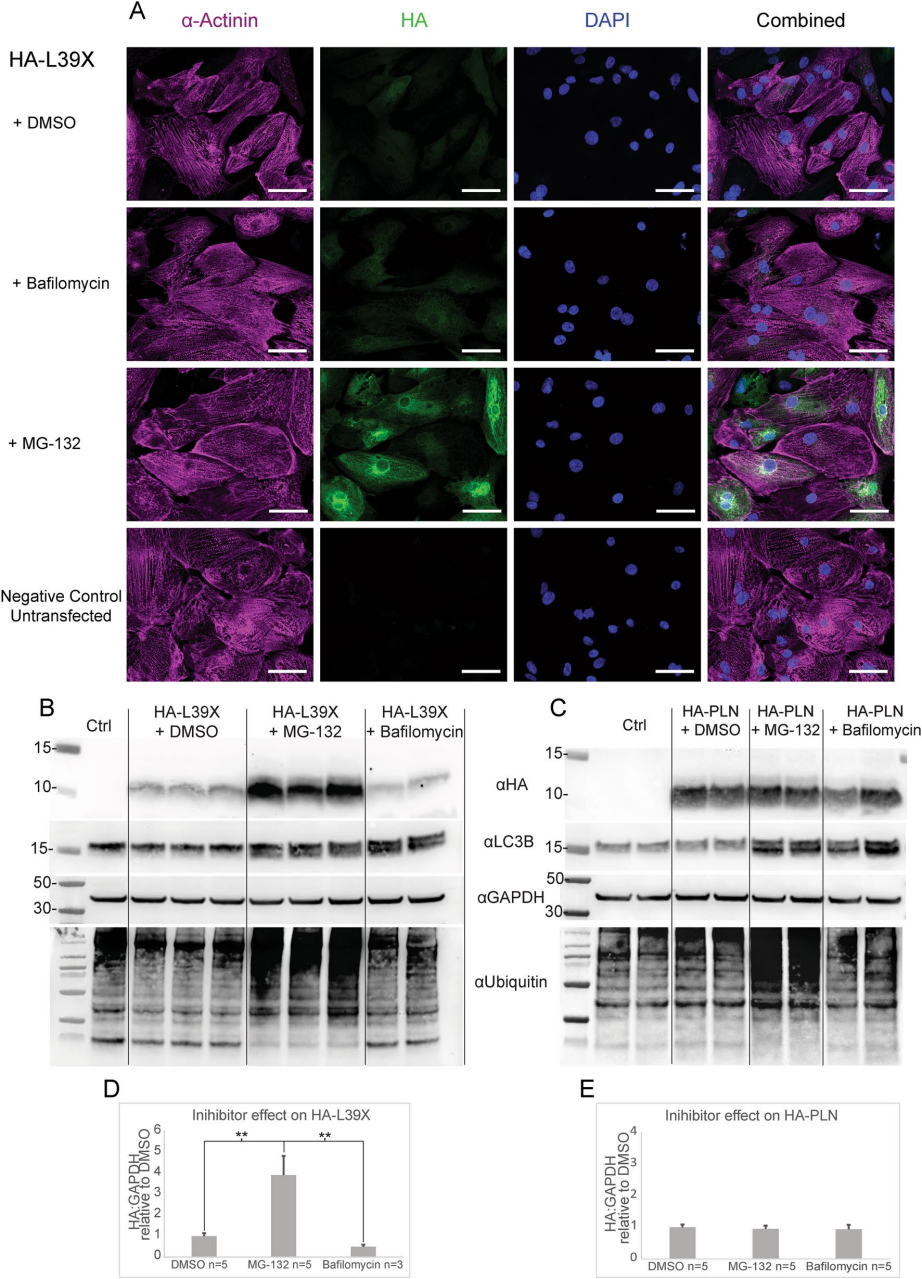
HA-L39X rescue assay using proteasomal and lysosomal inhibitors on transfected differentiated cardiomyocytes**. A** Immunofluorescence images of modHA-L39X transfected cardiomyocytes after a 5 h treatment with DMSO (first panel), 50 nM bafilomycin (second panel), 100 µM MG-132 (third panel) and negative untransfected control (fourth panel). α-Actinin (violet), HA (green), DAPI blue. Scale bars = 50 µm**. B**–**C** Western blot from modHA-L39X and modHA-PLN transfected cardiomyocytes after 5 h treatment with DMSO, MG-132 or bafilomycin. HA staining showing specific bands representing PLN monomers at ~ 10 kDa. GAPDH was used as loading control. **D**–**E** Bar graph illustrating the results from the Western blot experiments n = independent experiments. **P < 0.01

Further optimization of our imaging settings enabled us to identify a specific but faint HA signal in modHA-L39X transfected samples when comparing to untransfected cells (Fig. 3A).

The HA signal in the modHA-L39X transfected samples had a similar, but more diffuse perinuclear localization than in the modHA-PLN transfected samples (Figs. 2B, 3A). HA staining of Western blots from denatured cell lysates showed an HA specific band at approximately 10 kDa which represents PLN’s monomeric conformation (Fig. 3B).

As the above data showed evidence of the presence of our HA-L39X protein, we next sought to determine if we could rescue the signal by combining our overexpression assay with protein degradation inhibitors.

We selected two well established and specific chemical inhibitors. Bafilomycin, a lysosomal inhibitor that blocks vesicular fusion by inhibiting the proton pumps induced acidification, and MG-132, a proteasomal inhibitor that blocks the chymotrypsin-like activity of the proteasome (Goldberg 2012; Yoshimori et al. 1991). Cardiomyocytes treated with 50 nM of bafilomycin for 5 h showed increased expression of an autophagosome marker LC3B-II and a strong accumulation of cytoplasmic vesicles after 24 h (Fig. 3B–C and Additional file 1B, E). On the other hand, ubiquitin accumulation was observed in cells treated with 100 µM of MG-132 for 5 h (Fig. 3B–C and Additional file 1E).

Day 29 cardiomyocytes were transfected with 500 ng of HA-L39X modRNA, as done previously. At 19 h post-transfection, cells were treated with either DMSO, bafilomycin or MG-132 for 5 h (Fig. 3A and Additional file 1A). HA-L39X transfected cells treated with 100 µM MG-132 showed a strong increase in HA signal in both immunofluorescence and Western blots (Fig. 3A–B). Indeed, the HA signal was fourfold higher than that found in DMSO-treated samples after normalization to loading control (*P*-value < 0.01) (Fig. 3B, D). In contrast, treatment of the cells with 50 nM bafilomycin showed no signal increase in either immunofluorescence or Western blot assays (Fig. 3A–B, D). In contrast, Western blot analysis from HA-PLN transfected cells did not reveal significant increases in protein levels after treatment from either inhibitor (Fig. 3C, E). Thus, our results support that the L39X mutant protein is actively and specifically degraded by the proteasome.

### RNA sequencing indicates differential transcriptomic signature between PLN and L39X modRNA transfected cardiomyocytes

**Fig. 4:**
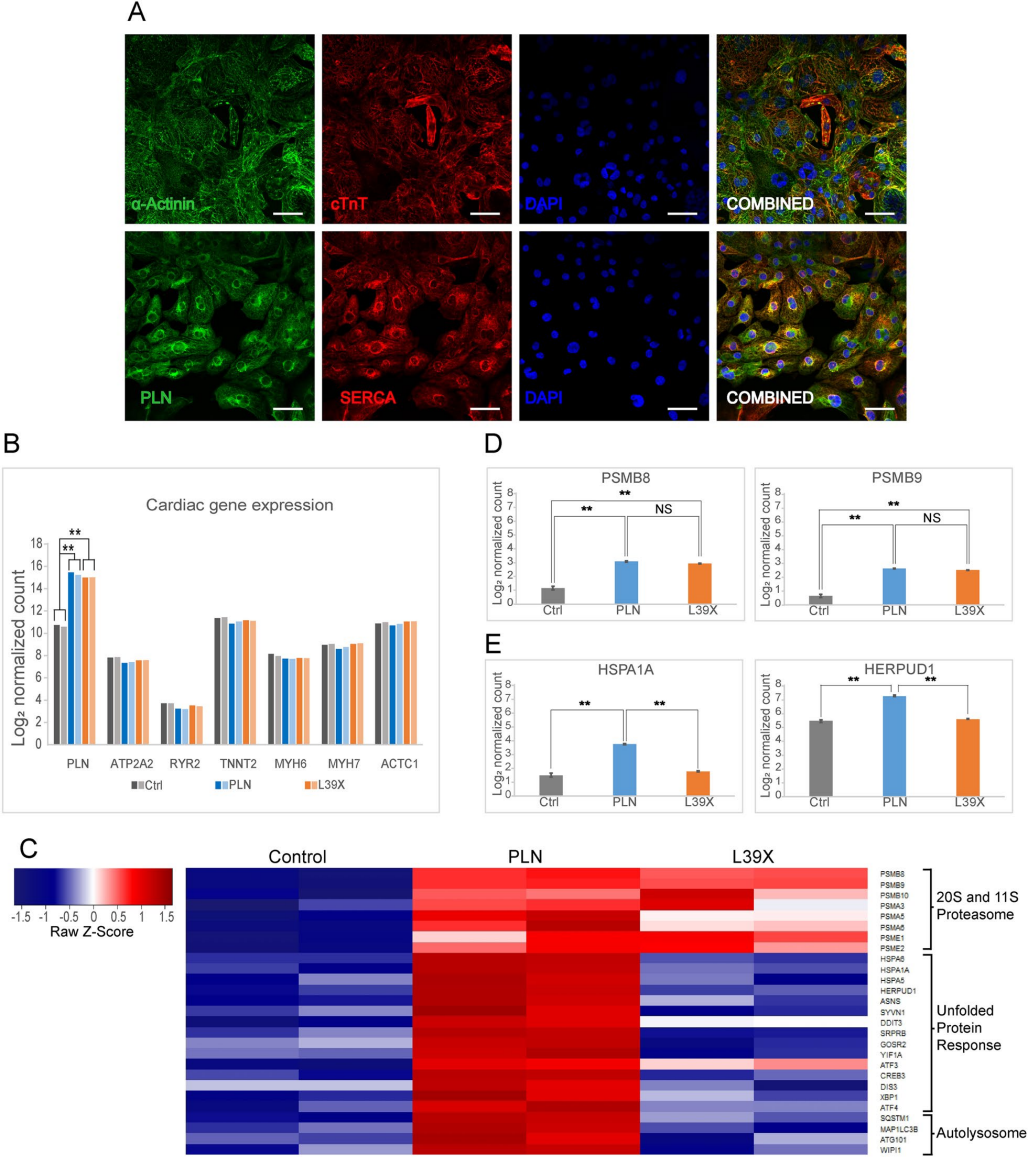
RNA sequencing analysis from 32 h modPLN or modL39X post-transfected cardiomyocytes. **A** Immunostaining of re-plated Day 30 cardiomyocytes showing complementarity (Top) of α-Actinin (green) and cTnT (red) and co-localization signals (Bottom) of PLN (green) and SERCA (red). Scale bar = 50 µm **B** EdgeR normalized gene expression of selected cardiac genes. Bar graphs illustrate duplicate experiments of control, modPLN and modL39X transfected cardiomyocytes. **C** Representative Heatmap from selected gene subsets, 20S proteasome (top), unfolded protein response (middle) and autolysosome (bottom). **D** and **E** Bar graphs representing EdgeR normalized counts of selected 20S proteasome genes (**D**) and unfolded protein response genes (**E**). **P < 0.01

Following the results shown in Fig. 3, we were interested in assessing whether acute L39X expression in cardiomyocytes was sufficient to alter the cells transcriptomic signature involved in protein degradation. We therefore transfected 500 ng of modPLN or modL39X modRNAs in Day 28.5 differentiated cardiomyocytes and proceeded to harvest total RNA from the cells at 32 h post-transfection (Additional file 1A). To limit the effect of well-to-well variation in cardiomyocyte differentiation, cells were collected, blended and re-seeded at day 23, prior to the performed experiments (Additional file 1A). By day 26 cells formed a monolayer and beat uniformly (Additional file 2). Pln, Serca2A, cTnT and α-actinin immunostaining from day 30 re-plated cells showed signal co-localization as expected for differentiated cardiomyocytes (Fig. 4A).

cDNA libraries for bulk RNA sequencing were generated after poly-A messenger RNA selection. All transfected samples had similar mRNA expression of common cardiac genes (*TNNT2*, *SERCA2A*, *RYR2*, *MYH6*, *MYH7* and *ACTC1*) indicating that the modRNA transfections did not impair the overall cardiomyocyte homeostasis (Fig. 4B). Both modPLN and modL39X transfected samples showed strongly upregulated PLN mRNA expression indicating similar transfection efficiency and mRNA stability (Fig. 4B).

Although we observed that modRNA transfection of a reporter gene did not significantly enhance gene expression (data not shown), GSEA analysis showed a strong and significant enrichment in proteasomal gene sets (hsa03050) in both modPLN and modL39X samples (Fig. 4C). Further analysis showed that the proteasome 20S catalytic core and 11S activator sub-units were significantly upregulated in modPLN and modL39X samples, illustrated by PSMB8 and PSMB9 normalized counts (Fig. 4C–D). Interestingly, PSMB8 which encodes for the iβ5 chymotrypsin like proteolytic site of the 20S proteasome, is known to be specifically inhibited by the MG-132 inhibitor which rescued our HA-L39X protein signal (Fig. 3B) (Goldberg 2012).

The modPLN transfected cells showed a strong and significant enrichment in unfolded protein response gene sets (R-HAS-381119), illustrated by HSPA1A and HERPUD1 normalized counts (Fig. 4C–E) and an upregulation of autolysosomal genes, which were not seen in the modL39X transfected samples (Fig. 4C–E). This suggests that the modPLN transfected cells are effectively synthesizing stably expressed PLN protein. The fact that neither proteasomal nor lysosomal inhibitors affected HA-PLN protein levels (Fig. 3C, E) suggests that the majority of the detected wild-type protein is stably expressed in these cells and not immediately targeted for degradation contrarily to L39X samples. This could in turn explain the lack of unfolded protein response seen in the modL39X transfected cells and the added upregulation of autolysosomal genes in the modPLN transfected cells. The autolysosomal pathway has been reported to constitute the normal degradation and recycling pathway of wild-type phospholamban proteins (Teng et al. 2015).

Thus, the L39X transcriptomic profile complements the observations made in Fig. 3. The proteasome rapidly and efficiently degrades the mutant protein and the observed trace amounts of protein are not sufficient to activate either the unfolded protein response or autolysosomal pathways.

In summary, we employed a modRNA acute overexpression assay and revealed the L39X nonsense phospholamban mutant generates a translated protein which in turn is rapidly degraded by the proteasome. We have further demonstrated the usage of modRNA as a tool to investigate translational and post-translational defects. To this end, optimization and flexibility of 3’UTRs, 5’UTRs, analogue capping and chemically modified nucleotides enable a rigorous control over the molecules’ stability, immunogenicity and translatability (Boo and Kim 2020; Chien et al. 2015; Pardi et al. 2018; Kaur et al. 2020). This control importantly enables focus on the mRNA’s message without the disturbance of other regulatory processes. The relatively high translatable yield and efficient delivery of modRNA into a large variety of cell types and organisms make it well-suited for the investigation of hard-to-detect proteins. Furthermore, modRNA could prove a useful tool to complement already popular modalities such as stem cells and genetic editing to further develop disease models.

Previous publications have shown that chronic impairment in protein degradation pathways can contribute to the development of cardiomyopathic phenotypes (Schlossarek et al. 2014; Gilda and Gomes 2017; Wang and Robbins 2014; Sarikas et al. 2005; Seeger et al. 2019). Further studies from patient derived iPSCs are needed to determine whether the chronic expression of L39X is sufficient to dysregulate proteolytic homeostasis in the human heart and further contribute to the pathophysiological outcome. In conclusion, we propose that in addition to the lack of functional PLN, the targeted degradation of the L39X protein by the proteasome could chronically contribute to the cardiomyopathic phenotype seen in human L39X carriers.

These findings emphasize additional important implications for other PLN mutations. Of note, a new nonsense PLN mutant has been recently reported in a Chinese population (Li et al. 2019). Carriers of this E2X mutant has shown a similar phenotype as observed in L39X carriers (Li et al. 2019). Determining whether this mutant protein is actively degraded through a similar or different degradation pathway could provide critical pathophysiological insights.

Interestingly, the discovery of new human PLN mutant variants and the improvement in gene therapy technologies has rekindled a once lost interest in phospholamban as a therapeutic target (Doevendans et al. 2019; Schmidt et al. 2001; Morihara et al. 2017; Spaeter et al. 2019; Chien et al. 2003). Calcium modulation has been a strong prospective therapeutic target in heart disease for more than two decades (Marks 2013; Jessup. 2011; Täubel et al. 2021; CAST 1989). Unfortunately, the initial failure from the CUPID phase 2 clinical trial, which consisted of an adeno-associated virus based SERCA overexpression therapy, put a hold on further assessments (Jessup. 2011; Lyon et al. 2020). At present, downregulation of phospholamban is being assessed as a potential therapy against heart failure and as a prospective gene therapy in R14del phospholamban mutant carriers (Doevendans et al. 2019; Schmidt et al. 2001; Morihara et al. 2017; Spaeter et al. 2019).

Notably, in contrast to homozygous carriers, the majority of heterozygous L39X (and E2X) carriers develop mild cardiomyopathy or no symptoms at all (Haghighi et al. 2003; Li et al. 2019). This implies that there is a certain human tolerance to chronic phospholamban downregulation regardless of the added hypothetical negative effect from this mutant’s chronic degradation. Taken together, our findings offer novel strategies for deciphering the molecular mechanisms of mutations associated with inherited cardiomyopathies.

## Conclusion

In this report we show compelling proteomic and transcriptomic evidence that the proteasome is responsible for the rapid degradation of the cardiomyopathic phospholamban null mutant. Further studies will be needed to understand the effects of chronic expression of the L39X mutant in humans. Our findings contribute to our understanding of the pathophysiology of L39X induced cardiomyopathy and to the development of potential new therapies.

## Supplementary Information

**Additional file 1: A** Overview schematic of performed experiments. **B** Bright field pictures from Day 30 cardiomyocytes treated with DMSO, 100 µM MG-132 or 50 nM bafilomycin for 24 h. Vacuole accumulation can be seen in bafilomycin treated cells. **C** Bioanalyzer data showing high RNA purity of all modRNA constructs synthesized in vitro. **D** Western blot from modHA-PLN and modHA-L39X (top) and transfected modHA-PLN-T2A-mCherry and modHA-L39-T2A-mCherry (bottom) Day 30 cardiomyocytes. HA staining showing 10 kDa band representing PLN monomeric conformation and mCherry staining showing a ~ 27 kDa band representing mCherry protein. **E** Western blot from untransfected and modHA-L39X transfected Day 30 cardiomyocytes treated with DMSO, 100 µM MG-132 or 50 nM bafilomycin for 5 h. LC3B staining showing LC3B-II accumulation in bafilomycin and MG-132 treated samples and Ubiquitin staining showing an accumulation of poly-ubiquitin in MG-132 treated samples. GAPDH is shown as loading control.

**Additional file 2:** Bright field video of beating day 26 re-seeded cardiomyocytes.

## Data Availability

The datasets used and/or analysed during the current study are available from the corresponding author on reasonable request.

## Abbreviations

PLN: Phospholamban
SERCA: Sarco-endoplasmic reticulum calcium-ATPase 2a
WT: Wild-type
modRNA: Chemically modified mRNA
hESC: Human embryonic stem cell
UTR: Untranslated region
FPKM: Fragments per kilobase of transcript per million mapped reads
GSEA: Gene set enrichment analysis
