# Supplementary figures and images for: An mRNA assay system demonstrates proteasomal-specific degradation contributes to cardiomyopathic phospholamban null mutation
Source: Mol Med. 2021 Sep 8;27:102. doi: 10.1186/s10020-021-00362-8 (PMC8425124; doi:10.1186/s10020-021-00362-8)

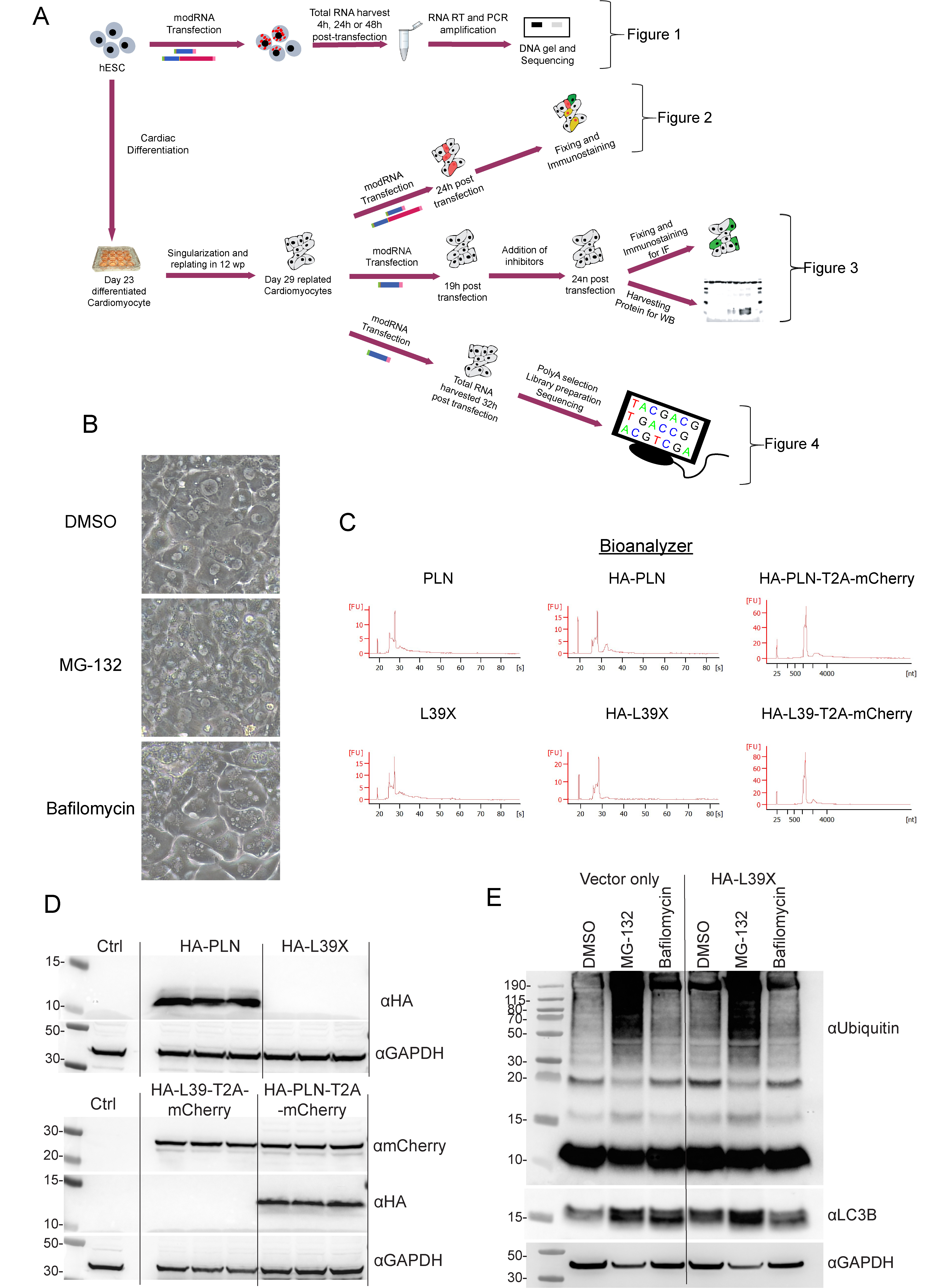

Supplement: Supplementary file 1 — Additional file 1: A Overview schematic of performed experiments. B Bright field pictures from Day 30 cardiomyocytes treated with DMSO, 100 µM MG-132 or 50 nM bafilomycin for 24 h. Vacuole accumulation can be seen in bafilomycin treated cells. C Bioanalyzer data showing high RNA purity of all modRNA constructs synthesized in vitro. D Western blot from modHA-PLN and modHA-L39X (top) and transfected modHA-PLN-T2A-mCherry and modHA-L39-T2A-mCherry (bottom) Day 30 cardiomyocytes. HA staining showing 10 kDa band representing PLN monomeric conformation and mCherry staining showing a ~ 27 kDa band representing mCherry protein. E Western blot from untransfected and modHA-L39X transfected Day 30 cardiomyocytes treated with DMSO, 100 µM MG-132 or 50 nM bafilomycin for 5 h. LC3B staining showing LC3B-II accumulation in bafilomycin and MG-132 treated samples and Ubiquitin staining showing an accumulation of poly-ubiquitin in MG-132 treated samples. GAPDH is shown as loading control. [file 10020_2021_362_MOESM1_ESM.jpg]
